# Segmentation of health-care consumers: psychological determinants of subjective health and other person-related variables

**DOI:** 10.1186/s12913-020-05560-4

**Published:** 2020-08-08

**Authors:** Sjaak Bloem, Joost Stalpers, Edward A. G. Groenland, Kees van Montfort, W. Fred van Raaij, Karla de Rooij

**Affiliations:** 1 Center for Marketing & Supply Chain Management, Nyenrode Business University, P.O. Box 130, 3620 AC Breukelen, The Netherlands; 2 Department of Biostatistics, Erasmus Medical Center Rotterdam, P.O. Box 2040, 3000 CA Rotterdam, The Netherlands; 3 Tilburg School of Social and Behavioral Sciences, Tilburg University, P.O. Box 90153, 5000 LE Tilburg, The Netherlands; 4 Janssen-Cilag B.V, PO Box 4928, 4803 EX Breda, The Netherlands

**Keywords:** Subjective health; person-centered segmentation; person-centric care, Demand-driven care, Acceptance, Perceived control

## Abstract

**Background:**

There is an observable, growing trend toward tailoring support programs – in addition to medical treatment – more closely to individuals to help improve patients’ health status. The segmentation model developed by Bloem & Stalpers [Nyenrode Research Papers Series 12:1–22, 2012] may serve as a solid basis for such an approach. The model is focused on individuals’ ‘health experience’ and is therefore a ‘cross-disease’ model. The model is based on the main psychological determinants of subjective health: acceptance and perceived control. The model identifies four segments of health-care consumers, based on high or low values on these determinants. The goal of the present study is twofold: the identification of criteria for differentiating between segments, and profiling of the segments in terms of socio-demographic and socio-economic variables.

**Methods:**

The data (acceptance, perceived control, socio-economic, and socio-demographic variables) for this study were obtained by using an online survey (a questionnaire design), that was given (random sample *N* = 2500) to a large panel of Dutch citizens. The final sample consisted of 2465 participants – age distribution and education level distribution in the sample were similar to those in the Dutch population; there was an overrepresentation of females. To analyze the data factor analyses, reliability tests, descriptive statistics and t-tests were used.

**Results:**

Cut-off scores, criteria to differentiate between the segments, were defined as the medians of the distributions of control and acceptance. Based on the outcomes, unique profiles have been formed for the four segments: 1. ‘Importance of self-management’ – relatively young, high social class, support programs: high-quality information. 2. ‘Importance of personal control’ – relatively old, living in rural areas, high in homeownership; supportive programs: developing personal control skills. 3. ‘Importance of acceptance’ – relatively young male; supportive programs: help by physicians and nurses. 4. ‘Importance of perspective and direction’ – female, low social class, receiving informal care; support programs: counseling and personal care.

**Conclusions:**

The profiles describe four segments of individuals/patients that are clearly distinct from each other, each with its own description. The enriched descriptions provide a better basis for the allocation and developing of supportive programs and interventions across individuals.

## Introduction

People’s health and the way in which they experience it are important components or indicators of their quality of life. In current practice, a range of innovations are providing an increasing number of opportunities to tailor medical treatment to individuals. This is referred to as ‘personalized medicine’ [1, 2]. Through this approach, the effectiveness and efficiency of treatments can be increased, enabling vital improvements to these individuals’ quality of life.

In addition to biomedical care, additional supportive care programs are provided in many therapeutic areas. Such programs aim at improving therapy adherence [3, 4], intend to stimulate self-management [5–7], or self-care [8, 9], or may be directed at improving life-style related aspects, such as physical fitness, or diet-related recommendations [10–12]. There is a general agreement that programs, to be efficient and effective, should ideally suit the needs, wants and wishes of individual health-care consumers [13, 14]. The trend toward tailoring programs more closely to individuals is aligned with developments in the field of personalized medicine. ICT developments will also help to ensure that supportive programs can be personalized further in the future [15, 16]. However, it is not always clear which programs are appropriate for which individuals [17–19]. The allocation and the effectiveness of supportive programs can be substantially improved by the application of techniques that help to increase the fit between the programs and the intended users on a systematic, scientific foundation. Which techniques are the most suitable?

### Segmentation

A generally accepted technique that is widely applied within marketing is consumer segmentation. Segmentation is an approach that aims at the differentiation of groups of individuals into segments [20] to align supply and demand and to facilitate the selection of the target groups [21, 22]. The principle of segmentation is directly applicable to the domain of health care in which the alignment of supply and demand is of vital concern [23, 24].

Most segmentation outcomes are empirically driven. In such instances, the segmentation criteria are based on practical considerations: a prior, post hoc, or data driven [25, 26]. Another approach is segmentation based on theoretical assumptions. This implies that the criteria for segmentation (e.g., behavior, attitudes, and beliefs) are based on/ or derived from a theoretical framework (e.g., ‘Adoption-diffusion theory’ of Rogers [27].

A major disadvantage of the empirical approach is that the use of empirical segmentation criteria may be suitable in a specific study, but problems arise when findings are generalized to other samples, other populations, or other situations or domains. This means that the segments are not stable over samples and over time. Another more fundamental problem is the fact that falsification is not possible: outcomes have no underlying theoretical framework but are exclusively based on a specific set of empirical data.

## Segmentation in health care

Segmentation as a basis for allocating services, products, and information to individuals is not commonplace in health care [28]. Segmentation procedures often use therapeutic domain or stage of development of a disease as criteria for differentiation (usually exclusively bio-medically oriented). The consequence of this approach is that depending on disease type, individuals, in addition to relevant medical treatment will receive the same additional service and support. At best, there may be some differentiation depending on the stage of the disease.

Studies that include psychological factors (as a base for segmentation) are rare (e.g. [29–35]). It should be mentioned that all these studies are empirically driven.

A promising model for segmentation in health care is the Bloem-Stalpers model [36]. The foundations of the model consist of two extensive research projects. The first study [37] aimed at the development of a theoretical basis of the concept of subjective health. This implied a clear definition, conceptualization, and operationalization of subjective health. The second study [38] focused on the identification of the main psychological determinants of subjective health.

In his study, Bloem [37] conceptualized subjective health as an idiosyncratic and holistic concept. This conceptualization of subjective health has led to the following definition: ‘Subjective health is an individual’s experience of physical and mental functioning while living his life the way he wants to, within the constraints and limitations of individual existence’. (p. 45). (For a further elaboration on the concept of subjective health, see [36, 37]).

The study by Stalpers [38] focused on the identification of the most important psychological determinants of subjective health: (a) acceptance of the disease and/or health level, and (b) perceived control over the personal health situation. He found that the determinants acceptance and perceived control are positively correlated, and that acceptance is a stronger determinant of subjective health than perceived control.

Acceptance is the feeling of the individual that his/her health status and the possible constraints on functioning are acceptable and fitting for him/her as a person. Perceived control is the belief of the individual that his/her health status, as perceived by him/herself, can be influenced or controlled by him/herself or by others. Higher levels of acceptance and perceived control are related to higher levels of subjective health (and well-being).

The concept of subjective health, and the two determinants (acceptance and perceived control) constitute the theoretical basis of the segmentation model. As higher levels of acceptance and perceived control are directly related to higher levels of subjective health, the two determinants serve as a basis for segmentation of health-care consumers. Acceptance and perceived control serve as the two dimensions of the model, and on each dimension, two levels of scores are proposed, high and low scores. This leads to four segments of health-care consumers, with each segment representing a specific type of individual.

**Fig. 1:**
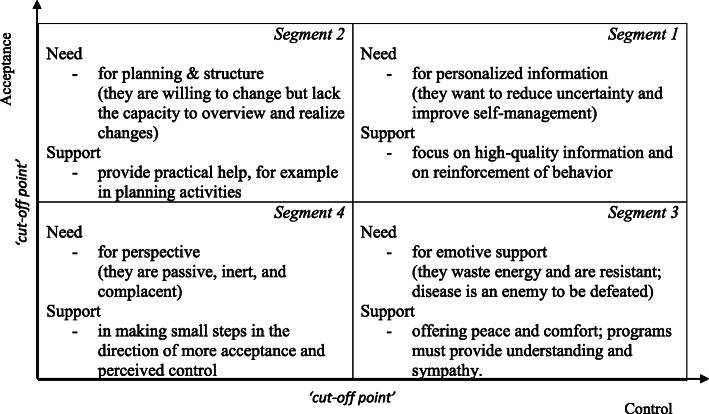
Segmentation model based on Bloem & Stalpers [36]

Based on underlying positions in terms of the two determinants, for each segment, generic psychological needs of individuals have been identified, theoretical [36, 39] and empirical [40]. It is important to note that the model is not designed for a specific patient population, but instead applies to a ‘general’ population. It is therefore based on a ‘cross-disease’ approach with a focus on individuals’ ‘health experience.’ The segments and the corresponding psychological needs are given in Fig. 1. For a full description of the model, see ([36], p. 10–11).

The model provides a solid framework for the segmentation of health-care consumers. First initial applications, where the framework is used, were focused on the development of a conversation approach for practice nurses [41], and the profiling of prostate cancer patients [40].

For the model to be of practical relevance, it needs further specification in terms of the following additions: (a) Further differentiation of the segments in terms the two determinants, and (b) Description and characterization of the segments in order to construct unique profiles for each segment. Consequently, the goal of the present study is twofold: the identification of criteria for differentiating between segments, and profiling of the segments of the Bloem-Stalpers model [36] with socio-economic and socio-demographic data.

In order to be able to differentiate between the segments of the model, it is essential that formal and final cut-off scores are identified on the two determinants. By determining these scores for a population of individuals (both healthy and non-healthy), a sound basis for comparison will be created for future studies. This study provides the basis for this.

To further specify the characteristics of the different segments, additional insights are needed. Over the last decades several studies have demonstrated relationships between subjective health and socio-economic and socio-demographic variables [42–44]. These relationships not only exist in the general population. Relations between person-related variables and subjective health have also been identified in various therapeutic areas [45, 46]. Both socio-economic and socio-demographic characteristics may be interpreted as specifications and articulations of the context within which an individual experiences subjective health, thus coloring and influencing this experience.

Therefore, a demonstration that the four segments differ in terms of socio-economic and socio-demographic variables will significantly contribute to the usefulness of the model in daily use. Advantages are that: (a) The segments can be described in more contextual detail; (b) Socio-economic and socio-demographic variables can be used to describe the context of the experience of subjective health of individuals, belonging to a certain segment; (c) These variables may serve as a source of inspiration for the development of additional support programs.

Please note that the study is focusing on the further characterization of the four segments. The study is not designed to investigate the effectiveness of supportive programs, and the study does not aim to establish the sensitivity and specificity of the classification (of four groups) to predict for example patient responses to ‘personalized medicine’ (targeted biomedical therapy).

Thus, the following research questions must be addressed: (a) How to identify formal and final cut-off scores on the determinants in order to allocate individuals to segments? (b) How is the Dutch population distributed over the four segments, given the cut-off scores? (c) How do the four segments differ in terms of socio-economic and socio-demographic variables? (d) Given the differentiation in terms of socio-economic and socio-demographic variables, which unique profiles of health-care consumers may be identified for the four segments?

## Method

### Procedure, participants and panel characteristics

In this study, a questionnaire design was used. All respondents were presented with the same questions. The data for this study was obtained by using an online survey that was given to a large panel of Dutch citizens. The panel consisted of individual members of the Dutch population who indicated that they were willing to participate in research projects. They were invited by email. Prior to acceptance, members were screened for their motivation for participating in research projects and their socio-economic, socio-demographic and residence characteristics. In addition, they were checked for duplicate panel memberships.

For all research within the research agency GfK, informed consent was treated as a formal procedure. Only respondents who declared that they had no objection to their responses being used for research were selected for the research. See GfK policy [47].

The data for this study was collected in the fall of 2012 as part of a comprehensive study into the health conditions, beliefs, values, and socio-economic and socio-demographic characteristics of individuals. During this period, the Dutch National Health system remained essentially the same in terms of internal structure and workings. Additionally, no societal volatility was observed from its participants during the past years at this point. Therefore, both the data and the outcomes of this study will describe the current circumstances in the Netherlands.

### Measuring determinants

Stalpers’ questionnaire [38] for measuring the determinants was used. The questionnaire was used for several reasons. The instrument had several advantages in comparison with other general health-related quality of life questionnaires. There was a clear conceptualization and operationalization, the concept of subjective health was based on a theoretical framework, the determinants were measured with a limited set of reliable questions (only six), and in addition, the model gave direction to the kind of supportive programs individuals needed [36, 37].

The items to measure the determinants were in keeping with the world of daily experience of the respondents. Stalpers [38] stated that: ‘The data [of the qualitative study] did provide indications about the semantic characteristics of the language used by individuals … when referring to subjective health and its psychological determinants. Insights into semantics contribute significantly to the quality of the items that were used to measure the concepts, specifically to content and construct validity’. (p. 100).

Three questions were asked on acceptance of personal health condition on a scale 1 = fully agree to 7 = fully disagree, thus creating quasimetric scales. From these questions, an acceptance scale has been formed:

1. ‘I am at peace with my health condition.’
2. ‘The way in which I am functioning physically and mentally, is acceptable to me.’
3. ‘I accept my health condition the way it is.’

Three questions were asked on perceived control of health condition on a scale 1 = fully agree to 7 = fully disagree, thus creating quasimetric scales. From these questions, a perceived control scale has been formed:

4. ‘I have the feeling that I have grip on my health condition.’
5. ‘My health condition is to a great extent in my own power.’
6. ‘I have a lot of influence on my health condition.’

These six questions were presented in a random order to the respondents.

### Statistical analyses

Factor analyses was used to summarize three questions regarding ‘acceptance of personal health condition’ to only one scale [48]. In the same way for every respondent a score corresponding to three items regarding ‘perceived control’ was calculated. Also, reliability levels (Cronbach’s alpha) of the item sets for the determinants perceived control and acceptance were calculated.

Next, a cut-off score was defined as the median value of the ‘acceptance of personal health condition’ scores. Another cut-off score was calculated as the median value of the ‘perceived control’ scores. Based on both cut-off scores four segments were constructed: Segment 1 (high score of acceptance; high score of perceived control), segment 2 (low score of perceived control, high score of acceptance), segment 3 (high score of perceived control, low score of acceptance), and segment 4 (low score of acceptance; low score of perceived control).

Finally, the socio-demographic characteristics of the patients in each segment were compared to those characteristics of the patients in other segments. By using t-tests it was analyzed whether the socio-demographic characteristics of patients in separate segments were statistically different.

## Results

### Sample

Upon checking the sample (a random sample, *N* = 2500) for the required quality demands, consisting of 2465 participants, the age distribution and the education level distribution in the sample were similar to those in the Dutch population. However, in the sample, there were more females (60.6%) compared to the Dutch population.

The characteristics of the sample (in terms of socio-demographic and socio-economic variables) were depicted in Table 1.

**Table 1:** Results of the socio-demographic and socio-economic variables of the sample

‘urbanization level’	52.2% urban	30.1% rural		
	(1 = > 2500 addresses per km^2^; 5 = < 500 addresses per km^2^)			
‘gender’	60.6% female	39.4% male		
‘age’	average of 46.7 years			
‘level of education’	17.9% low	59.0% average	23.0% high	
	(1 = no education; 7 = master’s degree)			
‘household size’	22.3% 1-person	44.0% 2-person	33.7% 3-person, >	
‘social class’	16.2% A	36.8% B	21.3% C	25.7% D-E (from high to low)
‘home ownership’	63.3% yes	36.7% no		
‘gross annual income’	average of €33.000			
	(1 = less than €12,000 per year;7 = more than €73,000 per year)			
‘religion’	45.9% yes	54.1% no		
‘strength of religious belief’	10.8% strong	30.9% average	60.3% not strong	
‘level of interest in politics’	10,6% strong	39.1% weak	50.2% not at all	
‘receiving informal care’	3.0% yes	97.0 no		

### Acceptance and perceived control scales

The explained variance of the factor analyses model regarding ‘acceptance of personal health condition’ was 75,25%. This meant that the scale, which was calculated as the average of the scores of the three items, represented those three items very well. In the same way a scale corresponding to three items regarding ‘perceived control’ was calculated. It turned out that the explained variance of this scale was 81,71%. So, the three questions regarding ‘perceived control’ could very well be represented by this single scale. The reliability levels of the item sets for the determinants perceived control and acceptance, as expressed in Cronbach’s alpha, were high, respectively with values of .87 and .88.

### Identification of cut-off points and construction of the segments

Cut-off scores were defined as the medians of the distributions of control and acceptance, respectively. As approximately one third of the sample consisted of males, as compared to an expected distribution of 50% of males in the population, an additional procedure had been carried out to check whether this characteristic had an effect on the values of these medians. To that end, the medians for control and acceptance in the population were calculated through the use of interpolation of the cumulative percentages for males and females, based on the starting point of an equal share of males and females. This procedure demonstrated that the cut-off points of the population-based medians were similar to the sample-based medians. Based on this outcome, the sample-based medians were accepted as true and reliable cut-off points.

The median value of perceived control is 5.36; the median value of acceptance is 4.96 (both on 7-point scales).

Based on the cut-off points four segments were then identified as follows:

- Segment 1: average score of acceptance > 4.96 and average score of perceived control > 5.36;
- Segment 2: average score of acceptance ≤4.96 and average score of perceived control > 5.36;
- Segment 3: average score of acceptance > 4.96 and average score of perceived control ≤5.36;
- Segment 4: average score of acceptance ≤4.96 and average score of perceived control ≤5.36.

The sizes of the four segments were: segment 1: 31.8%, segment 2: 17.4%, segment 3: 18.9%, and segment 4: 31.9%. The largest were the segments in which the levels of control and acceptance were both high (segment 1) or both low (segment 4). This indicated a positive correlation between perceived control and acceptance.

### Socio-economic and sociodemographic description of the segments

First, the scores on the categories of the socio-economic and socio-demographic variables were coded by the numbers 1, 2, 3, etc. (Table 2). Next, the average scores of the variables per segment were calculated and described in Table 2. By using t-tests was tested, whether the average scores of the variables were different (on a 5% level).

**Table 2:** Mean scores of the four segments and total sample on socio-economic and socio-demographic variables. Between brackets are the corresponding 95%-confidence intervals of the corresponding mean values

Characteristics	Segment 1	Segment 2	Segment 3	Segment 4	Total sample
Sizes of segments	(31.8%)	(17.4%)	(18.9%)	(31.9%)	
Urbanization	2.40	2.11	2.40	2.32	2.32
1 = low, 5 = high	(2.31–2.48)	(1.99–2.23)	(2.28–2.51)	(2.23–2.41)	(2.27–2.37)
Gender	1.52	1.67	1.58	1.67	1.61
1 = male, 2 = female	(1.48–1.55)	(1.62–1.71)	(1.53–1.62)	(1.64–1.71)	(1.59–1.63)
Age	43.96	51.23	44.99	48.01	46.71
in years	(42.74–45.18)	(49.62–52.85)	(43.51–46.47)	(46.91–49.11)	(46.05–47.38)
Level of Education	4.50	4.12	4.02	3.77	4.11
1 = low, 7 = high	(4.40–4.61)	(3.98–4.26)	(3.88–4.17)	(3.67–3.88)	(4.05–4.18)
Household size	2.44	2.43	2.42	2.32	2.40
Number of persons	(2.35–2.52)	(2.32–2.55)	(2.31–2.53)	(2.24–2.40)	(2.35–2.44)
Social class	2.60	2.53	2.36	2.15	2.40
1 = low, 5 = high	(2.52–2.67)	(2.44–2.63)	(2.26–2.46)	(2.07–2.23)	(2.36–2.44)
Home ownership	1.33	1.28	1.39	1.44	1.37
1 = yes, 2 = no	(1.29–1.36)	(1.24–1.32)	(1.34–1.43)	(1.41–1.48)	(1.35–1.39)
Gross annual income	4.01	3.97	3.84	3.54	3.82
1 = low, 7 = high	(3.87–4.15)	(3.79–4.15)	(3.65–4.03)	(3.40–3.67)	(3.74–3.90)
Religion	1.59	1.47	1.55	1.53	1.54
1 = yes, 2 = no	(1.55–1.67)	(1.42–1.52)	(1.51–1.60)	(1.49–1.57)	(1.52–1.56)
Strength of belief	2.39	2.74	2.38	2.71	2.55
1 = low, 7 = high	(2.24–2.53)	(2.54–2.95)	(2.20–2.56)	(2.57–2.86)	(2.47–2.63)
Interest in politics	2.34	2.41	2.43	2.42	2.40
1 = yes, 3 = no	(2.30–2.39)	(2.35–2.47)	(2.37–2.49)	(2.38–2.47)	(2.37–2.42)
Rec. informal care	2.00	1.98	1.98	1.93	1.97
1 = yes, 2 = no	(1.99–2.00)	(1.97–2.00)	(1.96–1.99)	(1.91–1.95)	(1.96–1.98)

Table 2 may be interpreted from two perspectives. On the one hand, perceived control and acceptance, taken in tandem, could be taken as a starting point in order to establish relationships with the various socio-economic and socio-demographic variables. On the other hand, for each of the segments the unique combinations of high versus low socio-economic and socio-demographic variables could be uncovered in order to sketch profiles of these combinations and relate them to the combined workings of perceived control and acceptance. Both approaches will be elaborated now. In the description, only significant differences will be interpreted.

Socio-economic and sociodemographic variables related to acceptance and perceived control:

Urbanization: urban versus rural: the combination of low levels of control with high levels of acceptance (segment 2) differed significantly from the other segments: in segment 2, a larger proportion lived in rural areas than in the other segments. One might speculate that this outcome points to a group of individuals who experience some difficulties in functioning adequately in terms of mental strength and disposition. Rural areas could provide the environment they need to screen themselves off from the outside world.

Gender: individuals with high levels of perceived control were predominantly male, whereas individuals with lower levels of perceived control were predominantly female. Notions of the traditional upbringing of boys and girls, in which the two are treated differently by their educators on the accepted level of expressed dominance toward others, was an explanation of this finding.

Age was related to level of perceived control, irrespective of level of acceptance: individuals in segments 1 and 3 were younger than individuals in segments 2 and 4. Younger individuals might have been better able to cope with demanding circumstances; this could explain their higher levels of perceived control.

Level of education: individuals who experienced both high levels of perceived control and acceptance (segment 1) differed significantly from individuals experienced other combinations of those determinants. Individuals in segment 1 were better educated than persons in the other three segments. Information is usually better understood by persons with a high level of education.

The opposite was also true: individuals who experienced both low levels of perceived control and acceptance (segment 4) differed significantly from individuals with other combinations. Individuals in segment 4 had a lower education than subjects in the other three segments. Education might have been widened the horizon and allowed for a broader view of life, while that might not have been the case for individuals with less education.

Household size: no differences were found between levels of acceptance and perceived control for household size.

Social class: individuals who experienced both low levels of perceived control and acceptance (segment 4) differed significantly from other segments. Individuals of segment 4 belonged to lower social classes than individuals of the other segments. Social class was correlated with level of education and income. Being connected to more affluent and better educated friends and family could have lead to a broader and relativistic perspective on life.

Home ownership: individuals who experienced both low levels of perceived control and acceptance (segment 4) differed significantly from other segments. Individuals of segment 4 were low in ‘home ownership’ compared to individuals of the other segments. Home ownership was correlated with level of education, social class and income.

High income earners: there was a clear tendency that individuals with higher levels of acceptance had a higher income than individuals with lower acceptance. As income level, social class, and home ownership were correlated, the same line of reasoning mentioned earlier applied. The costs of a treatment or information was less of a problem for high income earners.

Religion: the data suggested a relationship between level of perceived control and religion. Individuals with a low level of perceived control tended to be more religious than individuals with a high level of perceived control. There was a significant difference between segments 1 and 2. Scores on perceived control of the other two segments were in the expected direction. An explanation could be that religious individuals tend to believe that control over their life is external to themselves.

Strength of religious belief: as for religion, there seemed to be a relationship between level of control and strength of belief, with individuals higher on perceived control had low levels of religious belief than people low in perceived control. Again, there was a significant difference between segments 1 and 2, while scores of the other segments in the expected direction. Religious individuals tended to believe that control over their life was external to themselves.

Level of interest in politics: no relationships were found between levels of acceptance and perceived control with interest in politics, except that segment 1 is less interested in politics than the other segments.

Receiving informal care: individuals who experienced both low levels of perceived control and acceptance (segment 4) differed significantly from the other segments. Individuals in segment 4 were more likely to receive informal care than individuals in the other segments. The number of respondents who had received informal care is very low (3%).

### Profiles of the four segments

Based on the previous results, unique profiles of healthcare consumers for each of the four segments could been determined. To that end, the segment descriptions of the Bloem-Stalpers model [36], as presented in Fig. 1, were combined with the unique and specific combinations of socio-economic and sociodemographic characteristics that had emerged from the analyses. Table 3 shows these profiles. For each segment the socio-economic and socio-demographic characteristics were only mentioned if these characteristics were statistically different compared to the characteristics of the other segments.

**Table 3:** A contextual description (profile) of the four segments, based on socio-economic and socio-demographic variables

Segment	Contextual description
1. High control, high acceptance	male, young, high level of education, high social class, high home ownership, high gross annual income, not religious, low strength of believe.
2. Low control, high acceptance	non-urban, female, old, high social class, high home ownership, high gross annual income, religious, high strengths of believe.
3. High control, low acceptance	male, young
4. Low control, low acceptance	female, low level of education, low social class, low home ownership, low gross annual income, high strength of believe, receive informal care

#### Profile of Segment 1: Importance of self-management

Given their profile, individuals in this segment tried to manage their own lives. Consequently, support programs for this segment must provide high-quality information and reinforcement of behavior that would improve or maintain level of subjective health. Friends and family could support the individual. People in this segment were relatively young, had a high level of education, high social class, high income, were not religious and were not receiving informal care. Considering the education level and financial situation of these individuals, complexity of the supportive programs and costs involved were not a real issue. Information could be provided in printed media and on the Internet. People in this segment were likely to find and to understand this information.

#### Profile of Segment 2: Importance of personal control

Given their profile, individuals in this segment were able to internalize their health situation. However, they tended to attribute control over their life externally to others. This could explain the higher prevalence of individuals with a religious orientation in this segment. These individuals could benefit from supportive programs that help to develop personal control skills. Those programs would strengthen the individual’s confidence in the ability to exert control over their personal health condition. Persons in this segment were relatively old, high social class, living in rural areas, and were high in ‘home ownership’.

#### Profile of Segment 3: Importance of acceptance

Individuals in this segment had high personal control and low acceptance. They had difficulties living their lives with poor health. They were ‘fighters’ and perceived a disease or other health problems as personal ‘enemies’ to be defeated. They might have perceived good health as a ‘normal’ and required condition of life. They might even have thought to possess the ‘right’ to be healthy. If they were unhealthy, they required to be helped by physicians and nurses to get their health back. People in this segment were relatively young and male.

#### Profile of Segment 4: Importance of perspective and direction

Individuals in this segment lacked perspective. They were unable to internalize and accept their health condition. In addition, they were unable or unwilling to recognize the effectiveness of their own efforts to improve their health. To have changed the complacent situation of those individuals, support programs must have center on providing future perspective and efficacy. This might have been realized by setting attainable goals. These people needed personal counseling taking the individual by the hand, helping him/her to make (small) steps in the direction of more acceptance and more perceived control, and reinforcing desired and beneficial behavior. Effectiveness of support programs might have been enhanced by involving the individual’s social environment as a reinforcer of desired behavior. People in this segment were largely female, had a low level of education, low income, and low social class, were less likely to be ‘homeowners’, and ‘more’ likely to have received informal care. Print media and information on the Internet were less likely to reach and affect them. Counseling and personal care were needed to improve their subjective health.

## Discussion

This study used a sample of the general Dutch population. The proposed Bloem-Stalpers model applies to the whole population, not only to the chronically and temporarily ill. The two criteria, acceptance and perceived control, are useful and insightful in the formation of four health-care segments.

This study has produced a segmentation structure that is optimized in the domain of the subjective health experience of individuals. No limitations have presented themselves in the process of research and analysis. Therefore, the findings demonstrate without restrictions that in the empirical world of individuals and patients, four basic postures regarding the experience of subjective health exist. These basic postures are generic in nature and they shape the individual responses to ‘sickness and health’.

Persons with a high level of acceptance (segments 1 and 2) are likely to have their health condition internalized and accepted. This is a realistic pre-condition for planning, structuring and improving their lives according to their physical and mental possibilities. They need relevant and personalized information as input for the options and possibilities they (still) have. The Social Web itself provides many sources of information that can be used to extract information for personalization [49]. They are more likely to adhere to medicine treatments using apps [50], to follow medical advice, and, in this way, are more successful in improving their health. Especially in segment 1, people have a high level of education and income.

Persons with a low level of acceptance (segments 3 and 4) are likely to fight against a poor health condition. They will not ‘surrender’ and perceive a disease as an enemy to be defeated. They spend most of their efforts and energy in fighting and not in improving their condition, especially people in segment 3. This is not a realistic starting point for self-insights and self-management. Programs should offer peace, comfort and a realistic cognitive and emotional basis for self-knowledge and efficacy. Atkins et al. [51] present a framework how to change (health) behavior (see also [52]).

Persons with a high level of perceived control (segments 1 and 3) feel that they are masters of their own fate. They are likely to organize their lives personally and take measures if needed. If well-informed about their condition thus may be a successful strategy to improve their personal condition. People in these segments are often male, urban and non-religious.

Persons with a low level of perceived control (segments 2 and 4) feel that they are ‘victims’ of their situation and dependent on others and circumstances and are less able to manage their own situation. They need help with planning and personal guidance, and even hope for a better future (segment 4 – to improve self-efficacy health [53], and self-management behaviors for improving self-management [54]. People in segments 2 and 4 are very dissimilar: urban versus non-urban, high versus low social class, homeowner versus renter, high versus low income, religious versus non-religious, respectively. This means that acceptance may be a different characteristic for well-to-do versus poor respondents. As an example, Aldoory et al. studied the use of text messaging to disseminate health information to rural low-income mothers [55], and Hardman, Begg & Spelten assess the moderating effect of socioeconomic status on self-management support (SMS) interventions [56].

### Limitations

The sample of this study was drawn from the general population, with different degrees of health, and provides insights on two determinants of subjective health: acceptance and perceived control. The segmentation outcomes may be different for a sample of non-healthy persons and for samples of patients with specific diseases. These patients are more involved with and concerned about their disease than the general population. This study is a starting point for a series of studies with samples of persons with specific diseases and even stages of specific diseases (COPD, cancer, heart problems, etc.).

With this study we will know which information to give to patients of different segments. We should also know more about the media that can be used: written information in an app, internet or leaflet; video examples with interviews of patients on acceptance and perceived control; face-to-face contacts and consolation; and Q&A (questions and answers) options. Secondly, some patients may handle this themselves, independently, whereas others need personal counseling and assistance. How frequently should patients be contacted and supported? May patients be contacted as a group? Can patients help each other? Many questions requiring other studies on the optimization of support programs.

The description of the segments has been done with socio-economic characteristics (Table 2). The description of the segments may be enriched with values and lifestyle. Religion is such a value system, and there are other value systems and lifestyles related to political opinions, ecology, diversity, concerns about medical and social inclusion and exclusion, as the corona crisis shows for a number of countries. For future studies, values and lifestyle will provide a richer description and understanding of the segments.

Another limitation is the one-shot approach of this study. Monitoring patients over time may provide insights how subjective health develops over time during an illness or a medical treatment.

As a conclusion, future studies should be more specific on health and diseases, should be broader on descriptions, and, if possible, should monitor patients over time.

### Further research

A next step would be a further differentiation between types of chronical disorders, such as those in the areas of oncology, or diabetes. An empirical question that needs to be addressed here, is the question how individuals of different diseases are distributed over the four segments. Again, it is expected that the treatment will be more refined and more precise by using the model, because the reactions of the patient to the specific disease may be predicted from the specific profile of the patient. As a result, the treatment can be tailored to the patient’s needs. It can also be assessed whether levels of acceptance and personal control differ between diseases. Four segments can be formed, but the size of these segments may differ for different diseases.

In future studies, we cannot only differentiate between the four segments in terms of socio-economic characteristics, but also in psychographic characteristics, such as values and lifestyle [57, 58]. We expect that the four segments also differ in values and lifestyle. Values and lifestyle are expected to have closer connections to acceptance, perceived control and subjective health than socio-economic characteristics.

## Conclusion

The profiles, as presented above, provide segments of individuals/patients that are clearly distinct from each other, each with their own identity. At the same time, each type or segment, shows a set of defining characteristics that are consistent with each other, and make sense as a distinctive whole. When this information is made available to a medical officer, the treatment can be geared to the psychological make-up of an individual patient, and consequently be more effective. The segmentation provides an improved basis for the allocation of supportive programs and interventions across individuals. Beside this, additional information for each segment helps policy makers to optimize existing or to develop new supportive health programs – programs addressing the specific needs of these different patient groups. It also provides information on the use of media and approaches (print, Internet, personal counseling) in supportive programs. Further research is required to determine whether this approach will help to ensure that the better targeted allocation of supportive programs will lead to an improved effectivity of patient support programs and a higher cost efficiency.

## Data Availability

The data that support the findings of this study are available from GfK but restrictions apply to the availability of these data, which were used under license for the current study, and so are not publicly available. Data are however available from the authors upon reasonable request and with permission of GfK.
