# Correction to: Segmentation of health-care consumers: psychological determinants of subjective health and other person-related variables

**DOI:** 10.1186/s12913-022-07756-2

**Published:** 2022-04-11

**Authors:** Sjaak Bloem, Joost Stalpers, Edward A. G. Groenland, Kees van Montfort, W. Fred van Raaij, Karla de Rooij

**Affiliations:** 1 Center for Marketing & Supply Chain Management, Nyenrode Business University, P.O. Box 130, 3620 AC Breukelen, The Netherlands; 2 Department of Biostatistics, Erasmus Medical Center Rotterdam, P.O. Box 2040, 3000 CA Rotterdam, The Netherlands; 3 Tilburg School of Social and Behavioral Sciences, Tilburg University, P.O. Box 90153, 5000 LE Tilburg, The Netherlands; 4 Janssen-Cilag B.V, PO Box 4928, 4803 EX Breda, The Netherlands

**Correction to: BMC Health Serv Res 20, 726 (2020)**

**https://doi.org/10.1186/s12913-020-05560-4**

Following publication of the original article [1], the authors would like to correct an error in the **Statistical analyses** section under the heading **Method**.

The sentence currently reads:

segment 2 (low score of acceptance; high score of perceived control), segment 3 (high score of acceptance; low score of perceived control)

The sentence should read:

segment 2 (low score of perceived control, high score of acceptance), segment 3 (high score of perceived control, low score of acceptance)

The original article [1] has been corrected.
